# A Fluorine-Functionalized Tb(III)–Organic Framework for Ba^2+^ Detection

**DOI:** 10.3390/molecules29245903

**Published:** 2024-12-13

**Authors:** Yang Zhang, Hua Tan, Jiaping Zhu, Linhai Duan, Yuchi Ding, Fenglan Liang, Yongshi Li, Xinteng Peng, Ruomei Jiang, Jiaxin Yu, Jianjiong Fan, Yuhang Chen, Rimeng Chen, Deyun Ma

**Affiliations:** 1 College of Chemistry, Guangdong University of Petrochemical Technology, Maoming 525000, China; 2 College of Life Science, Zhaoqing University, Zhaoqing 526061, China; 3 School of Food and Pharmaceutical Engineering, Zhaoqing University, Zhaoqing 526061, China; 4 Zhangjiang Institute for Food and Drug Control, Zhanjiang 524008, China

**Keywords:** Tb-MOF, fluorine-functionalized, luminescence sensor, Ba^2+^ cation, selective C_2_H_2_ capture

## Abstract

The development of lanthanide–organic frameworks (Ln-MOFs) using for luminescence sensing and selective gas adsorption applications is of great significance from an energy and environmental perspective. This study reports the solvothermal synthesis of a fluorine-functionalized 3D microporous Tb-MOF with a face-centered cubic (**fcu**) topology constructed from hexanuclear clusters (Tb_6_O_30_) bridged by fdpdc ligands, formulated as {[Tb_6_(fdpdc)_6_(*μ*_3_-OH)_8_(H_2_O)_6_]·4DMF}_n_ (**1**), (fdpdc = 3-fluorobiphenyl-4,4′-dicarboxylate). Complex **1** displays a 3D framework with the channel of 7.2 × 7.2 Å^2^ (measured between opposite atoms) perpendicular to the *a*-axis. With respect to Ba^2+^ cation, the framework of activated **1** (**1a**) exhibits high selectivity and reversibility in luminescence sensing function, with an LOD of 4.34665 mM. According to the results of simulations, compared to other small gas molecules (CO_2_, N_2_, H_2_, CO, and CH_4_), activated **1** (**1a**) shows a high adsorption selectivity for C_2_H_2_ at 298 K.

## 1. Introduction

Water has become increasingly polluted as a result of the rapid expansion of agriculture and industry. It is well known that harmful ions can cause irreversible harm to humans and aquatic life, and they are a major contributor to climate change [1]. Ba^2+^ ions are heavy metal ions that cause protein denaturation as well as enzyme inactivation in most human organs. Additionally, it is extremely difficult to remove Ba^2+^ ions from the human body. Therefore, it is crucial to develop a material that is capable of sensing Ba^2+^ ions with high specificity [2]. Ba^2+^ is traditionally detected using flameless atomic absorption scatterometry (ICP-AES, ICP-MS, ETAAS, NAA, and HPLC). However, these methods have several limitations, including sophisticated instrumentation, complicated pre-treatment procedures, and time-consuming procedures, which makes them inefficient for fast and easy determinations of Ba^2+^ [3]. Luminescent-based probes have garnered significant interest for sensing applications due to their selectivity, sensitivity, cost-effectiveness, visualizability, and rapid response [4].

There is no doubt that carbon dioxide causes some environmental problems, such as global warming. Therefore, it is vital that CO_2_ is captured and separated from gas mixtures in order to solve the aforementioned environmental issues, which continues to be a challenge for scientists [5]. As the simplest alkyne in the petrochemical industry and an important monomer in petrochemicals, C_2_H_2_ has been widely used in manufacturing industrial chemicals such as acetaldehyde, acetic acid, benzene, synthetic rubber, and synthetic fiber. C_2_H_2_ can be produced in industry by cracking hydrocarbons with oxygen or by combusting methane with oxygen, in which CO_2_ or some other impurity will be absorbed [6]. So, it is important to find suitable porous materials to selectively uptake C_2_H_2_ from the mixtures of C_2_H_2_/CO_2_ to obtain C_2_H_2_ with high purity.

Lanthanide–organic frameworks (Ln-MOFs) have garnered significant attention in recent decades because of their unique coordination modes, high coordination numbers, distinctive luminescence properties, and rapid response. Ln-MOFs have been widely used in gas storage/separation, catalysis, luminescent sensing, biomedicine, and so on [7,8,9]. Ln-MOFs generally consist of lanthanide ions/clusters and organic linkers. Lanthanide ions are recognized for their intrinsic Lewis acidity and notable optical properties from 4f-4f electronic transitions, potentially forming robust frameworks with distinct emission peaks. Meanwhile, as a result of the π-conjugated organic ligands, energy was transferred from the ligand-excited state to the lanthanide-excited state, increasing emission intensity [10]. The red and green emissions of Eu^3+^ and Tb^3+^ ions can be observed by the naked eye, making them great sensing materials. Furthermore, recent studies indicate that MOFs, featuring open metal sites and fluorine-decorated binding sites, exhibit significant potential for selective C_2_H_2_ sorption, characterized by high IAST selectivity and substantial C_2_H_2_ sorption heat [11,12]. The open metal sites are susceptible to both C_2_H_2_ absorption and water molecule attack. Excessive C_2_H_2_ binding energy increases the energy required for adsorbent regeneration.

It has been found that in addition to enhancing hydrophobicity, fluorine atoms may also influence the MOF structure in other ways. Small atom radius, high electronegativity, and low electric polarization of fluorine atoms allow them to create specific adsorption sites for sorbed molecules, resulting in an increase in gas absorption capacity [13]. Furthermore, fluorine-functionalized MOFs makes them more resistant to oxidation and light, improving the stability of these materials against environmental influences [14].

Based on the above considerations, the organic ligand 3-fluorobiphenyl-4,4′-dicarboxylic acid (H_2_fdpdc) containing the fluorine group was chosen to construct a bifunctional Tb(III)–organic framework, {[Tb_6_(fdpdc)_6_(*μ_3_-*OH)_8_(H_2_O)_6_]·4DMF}_n_ (**1**), with a face-centered cubic (**fcu**) topology built from hexanuclear clusters (Tb_6_O_30_) bridged by fdpdc ligands. Despite the presence of other metal ions, complex **1** is highly selective for the detection of Ba^2+^ in solution. Moreover, in order to better understand the role of OH groups and fluorine atoms in activated **1**, simulations of the adsorption isotherms of C_2_H_2_, CO_2_, N_2_, H_2_, CO, and CH_4_ at 298 K were conducted. As far as we know, although complex **1** has been reported by xue et al. and used for CO_2_ adsorption [15], **1** is the initial instance of lanthanide-based MOFs built from fdpdc ligands and used for Ba^2+^ sensing.

## 2. Results and Discussion

### 2.1. The Crystal Structure of Complex ***1***

**Figure 1:**
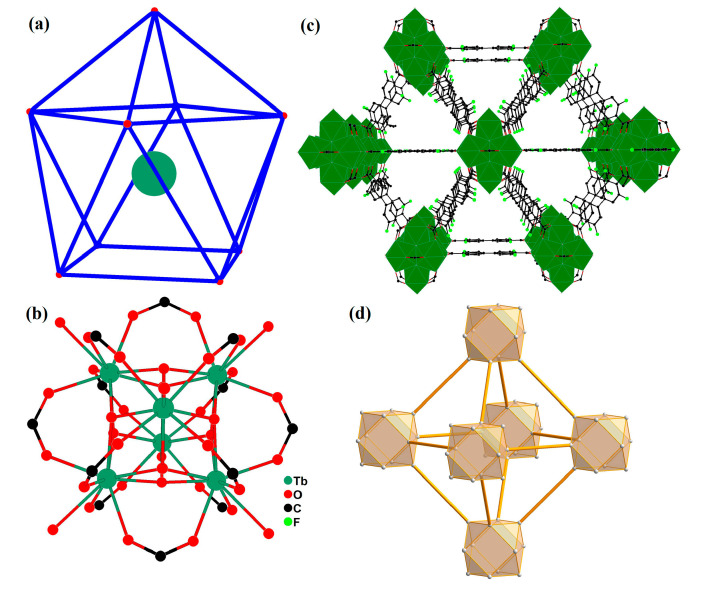
(**a**) Single-capped tetragonal prism of Tb(III) in **1**, blue line, configuration bar; red bullet, oxygen atoms; green circle, Tb. The coordination environment of Zn(II) ions in **1**. (**b**) Hexanuclear Tb_6_O_30_ unit of **1**. (**c**) View of the 3D framework packing perpendicular to the *a*-axis. (**d**) The **fcu** topology network of **1**.

Single-crystal X-ray diffraction analysis indicates that **1** crystallizes in the cubic space group Fm-3m and exhibits a 3D channel-type framework constructed by fdpdc ligands and hexanuclear clusters. Tb1 is nine-coordinated by four carboxylate oxygen atoms, four *μ_3_*-OH group, and one coordinated water molecule, and displays a single-capped tetragonal prism (Figure 1a). The bond lengths surrounding the Tb(III) centers (Td-O bond distances) are in the range of 2.3335(8)–2.652(11) Å, and the bond angles are in the range of 64.94(9)–139.80(5)°. As with other Tb(III) complexes containing oxygen-donating ligands, the values are consistent [16]. In the crystal structure of **1**, the fdpdc^2-^ anions act in a bridging-*μ_4_* mode to link four metal ions, whereas the OH group adopts the *μ_3_* bridging mode to connect three Tb(III) ions. The ten fdpdc^2-^ anions and eight *μ_3_*-OH groups link six Tb(III) ions to construct hexanuclear clusters (Tb_6_O_30_), which can be regarded as a supramolecular secondary building unit (SBU) or cluster node (Figure 1b). These units are interconnected through carboxylate groups of fdpdc^2-^ anions to generate a 3D framework with the channel of 7.2 × 7.2 Å^2^ (measured between opposite atoms) perpendicular to the *a*-axis (Figure 1c). Using PLATON software (for Windows Taskbar V1.10), the accessible volume in activated **1** (**1a**) after removing the guest molecules is 58.2%, with a potential void of 12,095.9 Å^3^.

In order to gain a deeper understanding of this framework, topology analysis is necessary [17]. In this structure, each hexanuclear unit (Tb_6_O_30_) acts as a 12-connected node, fdpdc^2-^ anions act as linker, and complex **1** can be represented as a face-centered cubic (**fcu**) net with a Schlafli symbol of {6^2^·8^2^·9^2^}_2_(6^2^·8}_4_(6^2^·9}_2_{6^3^·8·10^2^} (Figure 1d).

### 2.2. IR, TGA, and PXRD Analyses

A KBr pellet was used to record the FT-IR spectra. The IR spectra show an adsorption band at 3422 cm^−1^, which may be assigned to the ʋ(O-H) stretching vibrations of the water molecules. The adsorption bands observed at 2964 and 2902 cm^−1^ are attributed to the ʋ(C-H) vibrations of fdpdc anions. The strong and sharp bands at 1585 and 1400 cm^−1^ are associated with the asymmetric (C-O-C) and symmetric (C-O-C) stretching vibrations.

TGA was used to determine the thermal stability of compound **1**. Compound **1** was heated to 800 °C at a rate of 10 °C/min in an N_2_ atmosphere. The TG curve is shown in Figure S2. The TG curve for **1** shows the following three weight loss steps: The first, corresponding to the release of four guest DMF molecules and six aqua ligands, is observed from 50 to 300 °C (calcd.14.56%, obsd. 15.15%). The second, which corresponds to the release of eight OH groups, is observed from 300 to 450 °C (calcd. 4.95%, obsd. 5.20%). In the third, weight loss occurred above 450 °C due to the decomposition of the structure.

**Figure 2:**
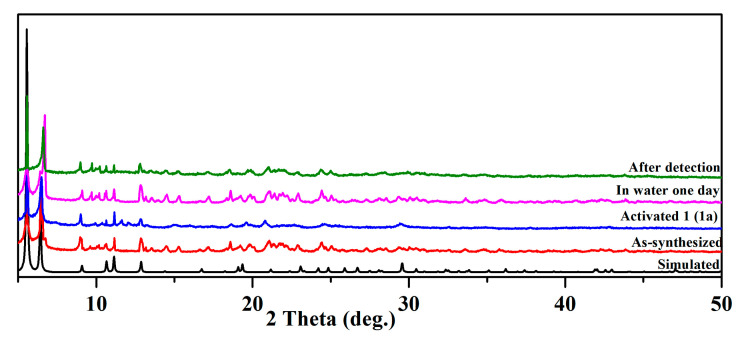
PXRD patterns of **1**.

At room temperature, bulk samples of **1** were also analyzed using X-ray diffraction. PXRD patterns for as-synthesized and activated **1** match well with their simulated counterparts, which indicates the same structure as the one solved with single-crystal diffraction (Figure 2). Although there were some peaks in the range of 9.5–10.5° after detection in water for one day, the positions and intensities of the major peaks (5.5° and 6.4°) were unchanged, indicating the stability of the structure. The water stability of compound **1** was evaluated by immersing 20 mg of the samples in water for one day. The PXRD patterns remained unchanged, indicating good water stability.

### 2.3. Sensing of ***1*** Toward Ba^2+^ Ion

**Figure 3:**
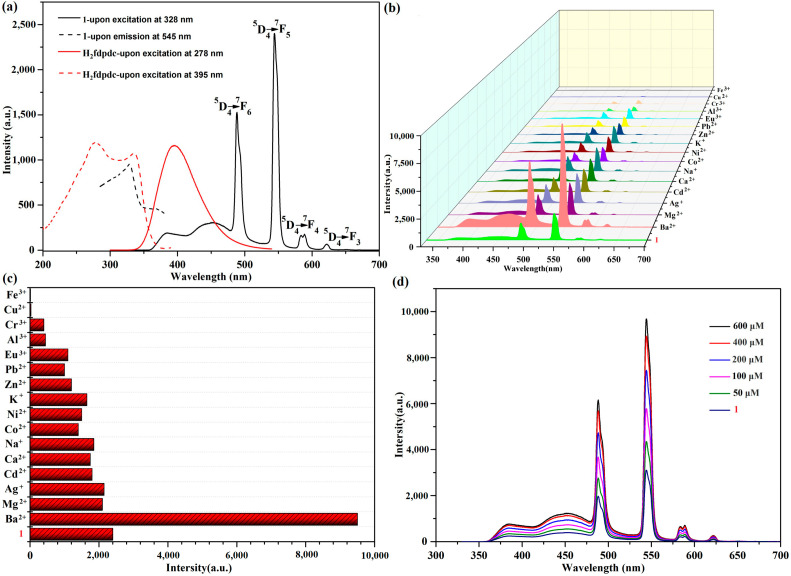
(**a**) The fluorescence excitation and emission of **1** and H_2_fdpdc ligand in solid state. (**b**) The cation-dependent fluorescence emission of **1**. (**c**) The luminescence intensity at 545 nm with the presence of different cations. (**d**) The luminescent emission spectra of **1** in Ba^2+^ solutions with different concentrations.

**Figure 4:**
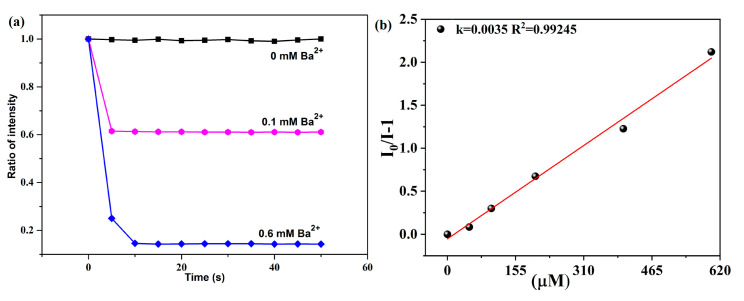
(**a**) The correlation between relative intensity and the addition of Ba^2+^ ion at different concentrations over time at 545 nm. (**b**) Calculated LOD of **1** in Ba^2+^ solutions.

Under ambient temperatures, we determined the luminescent property of **1a** (activated **1**) and H_2_fdpdc ligand using an excitation wavelength of 328 nm and 278 nm, respectively. Complex **1a** exhibits characteristic transitions of the Tb(III) ion at 489, 545, 588, and 622 nm, which correspond to ^5^D_4_→^7^F_n_ (*n* = 3–6) transitions, respectively (Figure 3a). When excited at 278 nm, the H_2_fdpdc ligand’s photoluminescence spectrum shows emission peaks at 395 nm (Figure 3a). It is probable that the emission band of the free ligand is caused by π*−π transitions. Upon excitation at 328 nm, the emission of **1** exhibits ligand-based emission between 300 and 400 nm and metal-based emission between 400 and 650 nm. It is important to note that H_2_fdpdc emission (395 nm) is absent in the luminescent spectrum of **1**, suggesting that the fdpdc^2-^ ligand can efficiently sensitize Tb^3+^ through the antenna effect. Compared to the free ligand H_2_fdpdc, the emission spectra of **1** exhibit a clear red shift. This occurrence can be explained by the interaction between lanthanide metal ions and ligands. It is interesting that complex **1** has a long fluorescence lifetime (τ = 120 ms at 545 nm) (Figure S3). To examine the potential of **1a** for sensing of cations (Ba^2+^, Mg^2+^, Ag^+^, Cd^2+^, Ca^2+^, Na^+^, Co^2+^, Ni^2+^, K^+^, Zn^2+^, Pb^2+^, Eu^3+^, Al^3+^, Cr^3+^, Cu^2+^, and Fe^3+^), the complex was immersed in different cations (chloride salts) aqueous solutions for luminescence studies. In order to evaluate the sensing performance, emission intensity at the peak of 545 nm was observed. The results revealed that photoluminescence (PL) is strongly dependent on the cations. As shown in Figure 3b,c, the addition of Ba^2+^ causes a significant enhancement of luminescence, while the addition of other metal ions causes the fluorescence intensity to weaken or quench. To further investigate the sensing performance of **1** for Ba^2+^ ion, the fluorescence titrations were performed by gradually adding the ions to a H_2_O suspension of **1**. Figure 3d demonstrates that emission was rapidly enhanced, with the Ba^2+^ concentration increasing from 0 to 600 μM. As a result, Ba^2+^ can be sensed selectively using compound **1**. The PXRD patterns of **1** after Ba^2+^ ion detection was collected, which reveals a similar profile with that of the as-synthesized one; the results indicated that the structure transformations/collapses were not responsible for changing luminescence signal. Furthermore, the reaction of luminescence to Ba^2+^ ion is instantaneous, reaching a maximum in only about 6 s at 545 nm (Figure 4a). The limit of detection (LOD) value was obtained based on the equation of LOD = 3σ/Ksv [18,19]. The result revealed that **1** has good selectivity for luminescent enhancement of Ba^2+^ ions, with a low detection limit (LOD) of 4.34665 mM in aqueous solution (Figure 4b). During the time-dependent intensity measurements, the whole fluorescence enhancement process takes only 6 s. The results suggest that **1** could be an efficient sensor for Ba^2+^ targets [19].

**Figure 5:**
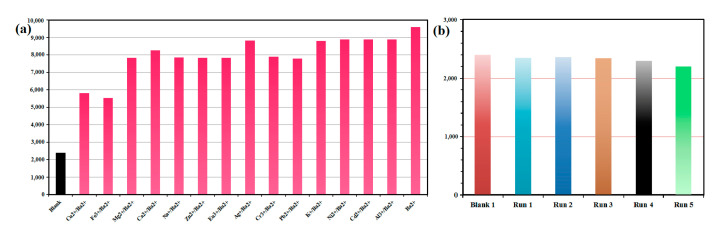
(**a**) Luminescence intensity of **1** at 545 nm dispersed in water with the addition of different metal ions. The concentration of interferential metal ions and Ba^2+^ ion are both 1 mM. (**b**) Luminescence intensity (545 nm) of **1** during five recycling.

One of the most important characteristics of sensors is their selectivity. Using mixed metal ions as an additional test, we evaluated the selectivity of **1** to Ba^2+^. As shown in Figure 5a, the enhancement efficiency of Ba^2+^ in the presence of other metal ions does not differ much from that in their absence. Despite Fe^3+^ and Cu^2+^ ions quenching luminescence significantly, the luminescence intensities remain higher than blanks. In the study, **1** was found to have high Ba^2+^ sensitivity and to be able to avoid interference from other metal ions.

We investigated the recycling performance of **1** as a Ba^2+^ sensor. The same recycling experiment was conducted five times (Figure 5b). The PXRD patterns and luminescence intensity of the recycled samples are in accordance with the blank of complex **1**.

The mechanism for **1**’s high sensitivity and selectivity to Ba^2+^ is discussed. There are many factors that influence the fluorescence intensity variations in MOFs, such as structural collapse, resonance energy transfer (RET), competitive absorption, and ionic exchange [20]. As one of these ions, Ba^2+^ exhibits such a high charge/size ratio that it is predicted to cause rupture of the C-H bond from the fdpdc ligands, resulting in hydronium ions (H_3_O^+^) [21], and the O atoms of *μ_3_*-OH group act as simple electron donor to Ba^2+^ ions through Lewis acid-base interactions. The luminescence enhancement of **1** toward Ba^2+^ may be caused by strong electrostatic interactions between Ba^2+^ ions and *μ_3_*-OH [22,23].

### 2.4. Sorption Performances

**Figure 6:**
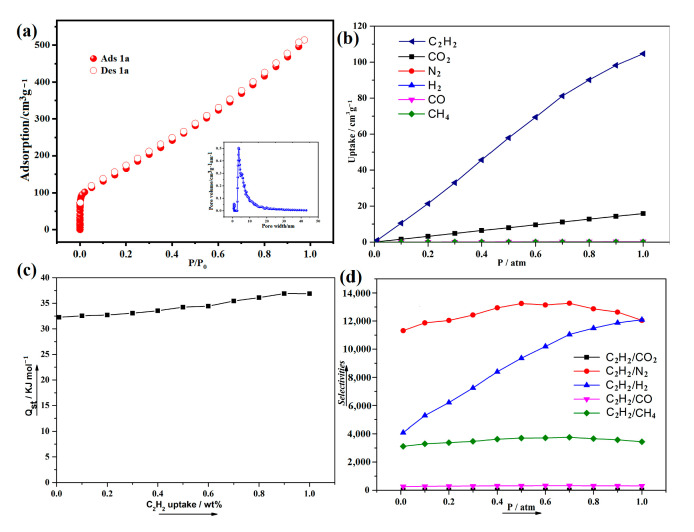
(**a**) N_2_ sorption isotherm of **1a** at 77 K (the inset shows the pore size distribution). (**b**) Room temperature adsorption isotherms of C_2_H_2_, CO_2_, N_2_, H_2_, CO, and CH_4_ based on the simulation. (**c**) Isosteric heat of adsorption of C_2_H_2_ for **1** calculated based on molecular simulation isotherms at 298 K. (**d**) Selectivities for C_2_H_2_ in the equimolar C_2_H_2_/CO_2_, C_2_H_2_/N_2_, C_2_H_2_/H_2_, C_2_H_2_/CO, and C_2_H_2_/CH_4_ at 298 K from the simulation.

We modified the outgassing temperature to 120 °C for 8 h to remove the guest molecules. At 77 K, N_2_ adsorption of **1a** was carried out and exhibits a type I isotherm (Figure 6a). The BET surface area of **1a** was calculated to be 1905 m^2^/g, which is in good agreement with the theoretical value (1986.63 m^2^/g). The Atom Volumes & Surfaces tool in MS software (v3.2) was used to calculate the theoretical specific surface areas of **1a**, utilizing N_2_ as the probe molecule. According to the NLDFT model, the pore volume and pore size width of **1a** are 0.52 cm³/g and 7.2Å, respectively.

In recent years, it has becoming increasingly common to use simulation to model the sorption properties of MOFs. A study of the adsorption properties of C_2_H_2_, CO_2_, N_2_, H_2_, CO, and CH_4_ has been conducted in **1a** (Figure 6b). C_2_H_2_ is preferentially adsorbable in **1a** due to the more intense dispersion interactions with the pores, as the pores of **1** are decorated by OH groups and fluorine atoms, and these groups point toward the center of the channels within complex **1**, enhancing the binding affinity between C_2_H_2_ and framework. We calculated the isosteric heat (*Q_st_*) of C_2_H_2_ adsorption based on the molecular simulation adsorption isotherm at 298 K. As shown in Figure 6c, *Q_st_* values range from 32.3–36.9 kJ·mol^−1^ for compound **1**. The *Q_st_* value for **1** at zero loading and 298 K is 32.3 kJ·mol^−1^. This value is higher than other reported fluorine-decorated MOFs, such as [In(TBOT)(2,3-FDA)](DEF)(H_2_O)_2_}_n_ (25.9 kJ·mol^−1^), UTSA-121 (22.6 kJ·mol^−1^), SIFSIX-3-Ni (25.9 kJ·mol^−1^), ELM-12 (25.4 kJ·mol^−1^), UPC-106 (19.0 kJ·mol^−1^), Iso-MOF-2 (17.4 kJ·mol^−1^), Iso-MOF-4 (27.8 kJ·mol^−1^), UiO-67-F8 (17.6 kJ·mol^−1^), UPC-200(Fe)-F-BIM (20.43 kJ·mol^−1^), UPC-200(Al)-F-BIM (18.9–20.5 kJ·mol^−1^), {(Et_2_NH_2_)[In(TBOT)(2,3-FDA)](DEF)(H_2_O)_2_}_n_ (25.9 kJ·mol^−1^), {[(CH_3_)_2_NH_2_]_2_[Dy_6_(m_3_-OH)_8_(FTZB)_6_(H_2_O)_6_](solvent)}_n_ (26.7 kJ·mol^−1^), and Cu-FINA-2 (12.3 kJ·mol^−1^) [24,25,26,27,28,29,30,31,32]. The findings demonstrate a significant interaction between C_2_H_2_ and **1a**. The introduction of fluorine atoms on the organic ligands and OH group can significantly enhance the H-bond interaction (C-H···F, C-H···O) [33].

Furthermore, the selective adsorptions of C_2_H_2_ from the equimolar C_2_H_2_/CO_2_, C_2_H_2_/N_2_, C_2_H_2_/H_2_, C_2_H_2_/CO, and C_2_H_2_/CH_4_ from 0 to 1 atm are illustrated in Figure 6d. Selectivities were calculated by using the thermodynamic parameter A/B = xA.yB/xB.yA, where x represents the adsorbed molar fraction and y represents the gas molar fraction of the compound. Obviously, C_2_H_2_ is more preferentially adsorbed than these gas molecules, including CO_2_, N_2_, H_2_, CO, and CH_4_, which may be due to the stronger dispersion interactions toward the pores of **1a**. Their pressure-dependent selectivity is similar, i.e., slightly/obviously decreasing with increasing pressure. It can be attributed to the packing effect that makes CO_2_, N_2_, H_2_, CO, and CH_4_ adsorption less significant, resulting in an obvious increase in C_2_H_2_ selectivity as pressure increases [34].

## 3. Materials and Methods

### 3.1. Materials and Physical Measurements

Reagent grade H_2_fdpdc ligand (98%) and Tb(NO_3_)_3_·6H_2_O (99.999% trace) were procured from Aladdin and used as received. Solvents involving DMF were obtained from Aladdin. The metal salts utilized in the sensing experiments were procured from Aladdin.

On a PerkinElmer 240 CHN elemental analyzer, C, H, and N were analyzed. KBr disks were used to measure infrared spectra with a Shimadzu IR-440 spectrometer. Thermogravimetric analyses (TGA) were performed using a Shimadzu DTG-60 simultaneous thermal analyzer under a nitrogen atmosphere with a heating rate of 10 °C /min. An AXS D_8_-Advance diffractometer with Cu-Kα (λ = 1.5418 Å) radiation was used to collect powder X-ray diffraction patterns (PXRD). At room temperature, an EdinburghFLS920 fluorescence spectrometer was used to measure the luminescence spectra and lifetime of crystalline samples. The experiments of N_2_ adsorption were carried out using a Quantachrome Autosorb-iQ facility.

### 3.2. Synthesis of {[Tb_6_(fdpdc)_6_(μ_3_-OH)_8_(H_2_O)_6_]·4DMF}_n_ (***1***)

3-fluorobiphenyl-4,4′-dicarboxylic acid (0.78 g, 3 mmol), Tb(NO_3_)_3_·6H_2_O (1.36 g, 3 mmol), DMF (5 mL), and H_2_O (5 mL) were added to a 23 mL Teflon reactor, and the resulting solution was heated (150 °C, 72 h). The mixture was cooled to room temperature at a rate of 5 °C/h. Light-yellow crystals were obtained in a yield of 67% based on Tb. Calcd for Tb_6_C_76_H_82_O_42_F_6_N_4_ (1): C, 30.0; N, 1.8; H, 2.7. Found: C, 31.9; N, 2.2; H, 2.6. IR (KBr pellet) (cm^−1^): 3422(vs, ʋ(O-H)), 2964(w), 2902(w), 1646 (m), 1585(s), 1510(m), 1400(vs), 1361(w), 1263(m, ʋ(C-F)), 1171(w), 1056(w), 1042(w), 844(w), 776(vs), 648(s), 586(m), 437(w) (Figure S1).

### 3.3. X-Ray Crystal Structural Determination

Complex **1** data were collected using a Bruker Apex II CCD diffractometer with a 50 kV and 30 mA MoK radiation source (λ = 0.71073 Å). The APEX II software (v2.0) was used to collect and reduce data [35]. Direct methods were used to solve the structure, followed by least-squares method on *F^2^* using SHELXTL [36]. Anisotropic displacement parameters were taken into account for non-hydrogen atoms, and the riding model was used to refine hydrogen atoms attached to carbon and oxygen. By applying SQUEEZE (PLATON), we removed diffuse electron density caused by poorly disordered guest DMF molecules [37]. Infrared, thermogravimetric, and elemental analyses were used to determine the formula unit of **1**. An analysis of the topology of the title compound was carried out using the TOPOS 4.0 software [17]. More detailed information can be found in the CIF file. Table 1 shows crystallographic data and refinement details for compound **1**. Table 2 presents selected bond lengths and bond angles.

### 3.4. Preparation of Ba^2+^ Aqueous Solution

Standard solution of BaCl_2_ (0.01 M) was obtained by dissolving BaCl_2_ (208.23 mg, 1.0 mmol) in 50 mL deionized water and then continuously adding deionized water to 100 mL. Different concentrations of Ba^2+^ solutions were obtained by diluting the standard solution (0.01 M) with deionized water (50 μM, 100 μM, 200 μM, 400 μM, and 600 μM).

### 3.5. Fluorescent Studies

An ultrasonic dispersion method was used to combine 5.0 mg of **1** with 1 mL of deionized water and disperse it. Then, different concentrations of Ba^2+^ solutions prepared in deionized water were added into the **1** original suspension (one solution for each suspension). Upon excitation at 328 nm, luminescence was observed. The homogeneity of solutions was maintained by stirring them continuously throughout the experiment. A triplicate of each experiment was conducted, and consistent results were obtained. After the experiment, we washed the samples three times with deionized water and dried them at 100 °C for 30 min.

### 3.6. Theoretical Simulations Section

Force field. CO_2_ was regarded as a rigid linear molecule with 0.116 nm C-O bond length. The LJ potential parameters of O atom (*σ_O_* = 0.305 nm and *ε_O_/κ_B_* = 79.0 K) and C atom (*σ_C_* = 0.280 nm and *ε_C_/κ_B_* = 27.0 K) in CO_2_ molecule were taken from the TraPPE force field [38]. Partial point charges are *q_O_* = −0.35e and *q_C_* = 0.70e. CH_4_ was represented by united-atom model (*σ* = 0.373 nm and *ε/κ_B_* = 148.0 K) [39]. N_2_ molecule was treated as a three-site model with three sites located at two N atoms and its center of mass COM (*σ_N_* = 0.331 nm, *ε_N_/κ_B_* = 36.0 K, *q_N_* = −0.482e and *q_COM_* = 0.964e) with the N-N bond length of 0.110 nm [38]. The SK model with three sites developed by Straub and Karplusa was employed to describe the CO molecule. Similar to N_2_, the SK model is operated based on the combination of three Lennard-Jones pair potentials and three partial point charges, which are located at the LJ centers (i.e., the carbon and oxygen atoms) and center of mass (COM) site (*σ_C_* = 0.383 nm, *ε_C_/κ_B_* = 13.18 K, *σ_O_* = 0.312 nm, *ε_O_/κ_B_* = 80.06 K, *q_O_* = −0.85e, *q_C_* = 0.75e and *q_COM_* = 1.60e). H_2_ molecule was regarded as a two-site LJ molecule as in our previous works (*σ_H_* = 0.272 nm and *ε_H_/κ_B_* = 10 K) [40]. To represent vdW interactions, the C_2_H_2_ molecule was treated as a two-site model, with the LJ positions located on the carbon atoms. The LJ parameters were originally proposed for the central CH groups of 2-butene [41], which have been successfully used in earlier MD studies of acetylene diffusion [42].

Monte Carlo simulation details. For the determination of single gas adsorption isotherms (C_2_H_2_, CO_2_, N_2_, H_2_, CO, and CH_4_) at 298 K, GCMC simulations were used. The material framework has been modeled as rigid in our simulations [43]. The simulation box contains 2 × 1 × 4 unit cells. A cutoff radius of 12.8 Å was applied to the Lennard-Jones interactions. Additionally, long-range electrostatic interactions with periodic boundary conditions were handled using the Ewald summation technique. For each state point, the number of steps in GCMC simulation was 2 × 10^7^, where the first 10^7^ steps were used to guarantee the equilibration, and the subsequent 10^7^ steps were used for sampling the desired thermodynamics properties [44]. The following is the equation for calculating the isosteric heat of adsorption, *Q_st_*:

(1) $$Q_{\mathrm{st}}=\;RT\;-\;\frac{<U_{ff}N>-<U_{ff}><N>}{<N^{2}>-<N><N>}-\frac{<U_{sf}N>-<U_{sf}><N>}{<N^{2}>-<N><N>}$$

where *R* is the gas constant, *N* is the number of molecules adsorbed, and < > indicates the ensemble average [45]. The first and second terms are the contributions from the molecular thermal energy and adsorbate–adsorbate interaction energy *U_ff_*, respectively, while the remaining term is the contribution from the adsorbent–adsorbate interaction energy *U_sf_* [46].

## 4. Conclusions

In summary, a dual-functional Tb-MOF has been successfully synthesized by using a fluorine-functionalized ligand strategy. Due to the fact that a fluorine-containing system was incorporated into the title compound, abundant F and O donor sites can be utilized for selective C_2_H_2_ binding. Complex **1** demonstrated high selectivity and sensitivity for the detection of Ba^2+^ in aqueous media through luminescence enhancement (turn-on), with a LOD of 4.34665 mM. Furthermore, in contrast to other small gas molecules, including CO_2_, N_2_, H_2_, CO, and CH_4_, **1** performs a highly selective adsorption of C_2_H_2_.

## Figures and Tables

**Table 1:** Crystallographic data of complex **1**.

Formula	C_84_H_42_F_6_O_35_Tb_6_	Z	4
Formula weight	2678.69	Density (g cm^−3^)	0.856
Cystal system	cubic	Limiting indices	−33 < h < 34, −34 < k < 29, −36 < l < 25
Space group	Fm-3m	Reflections collected/unique	25,533/19,248
Temperature (K)	100	R_int_	0.0231
Size (mm)	0.20 × 0.17 × 0.18	F(000)	5080
a (Å)	27.4995(2)	θ (°)	2.0800–29.4250
b (Å)	27.4995(2)	Goodness-of-fit on *F*^2^	1.057
c (Å)	27.4995(2)	*R* (*I* > 2*σ*)	*R*_1_ = 0.0276 w*R*_2_ = 0.0743
α = β = γ(°)	90	*R* (all data)	*R*_1_ = 0.0313 w*R*_2_ = 0.0775
V (Å^3^)	20,795.7(5)	Largest diff. Peak and hole (Å^−3^)	0.30, −0.17

**Table 2:** Selected bond length and angles of **1** (Å, °).

Tb1-O2	2.3335(8)	Tb1-O3	2.338(3)
Tb1-O6	2.652(11)	O2Tb1-O2	66.23(7)
O2Tb1-O3	76.94(9)	O2Tb1-O6	129.41(7)
O3Tb1-O3	129.88(18)	O3Tb1-O6	64.94(9)

## Data Availability

The detail crystallographic data of **1** are provided in CIF files.

## Supplementary Materials

The following supporting information can be downloaded at: https://www.mdpi.com/article/10.3390/molecules29245903/s1, Figure S1: IR spectra of **1**. Figure S2: TGA curves of **1** and activated **1** (**1a**). Figure S3: Luminescent lifetime for **1** in solid. CCDC: 2402714. Copies of this information may be obtained free of charge from the Director, CCDC, 12 Union Road, Cambridge, CB21EZ, UK (fax: +44 1223 336 033; e-mail: deposit@ccdc.cam.ac.uk or (http://www.ccdc.cam.ac.uk (accessed on 14 November 2024)) or are also available from the author on request.
