# Supplementary material for: A Fluorine-Functionalized Tb(III)–Organic Framework for Ba2+ Detection
Source: Molecules. 2024 Dec 13;29(24):5903. doi: 10.3390/molecules29245903 (PMC11677539; doi:10.3390/molecules29245903)
Supplement: Supplementary file 1 [file molecules-29-05903-s001.zip › molecules-3345908-supplementary.pdf]

Supporting Information

A Fluorine-functionalized Tb(III)-organic framework for Ba^{2+} detection

Yang Zhang [1], Hua Tan [1], Jiaping Zhu[1], Linhai Duan[1], Yuchi Ding[2], Fenglan Liang[2,*], Yongshi Li[3], Xinteng Peng[3], Ruomei Jiang[3], Jiaxin Yu[3], Jianjiong Fan[3], Yuhang Chen[3], Rimeng Chen[4], Deyun Ma[3,*]

[1] College of Chemistry, Guangdong University of Petrochemical Technology, Maoming 525000, P.R. China e-mail@e-mail.com

[2] College of Life Science, Zhaoqing University, Zhaoqing 526061, P. R. China

[3] School of Food and Pharmaceutical Engineering, Zhaoqing University, Zhaoqing 526061, P. R. China

[4] Zhangjiang Institute for Food and Drug Control, Zhanjiang, 524008, P. R. China

* Correspondence: mady@zqu.edu.cn and liangfl82@126.com

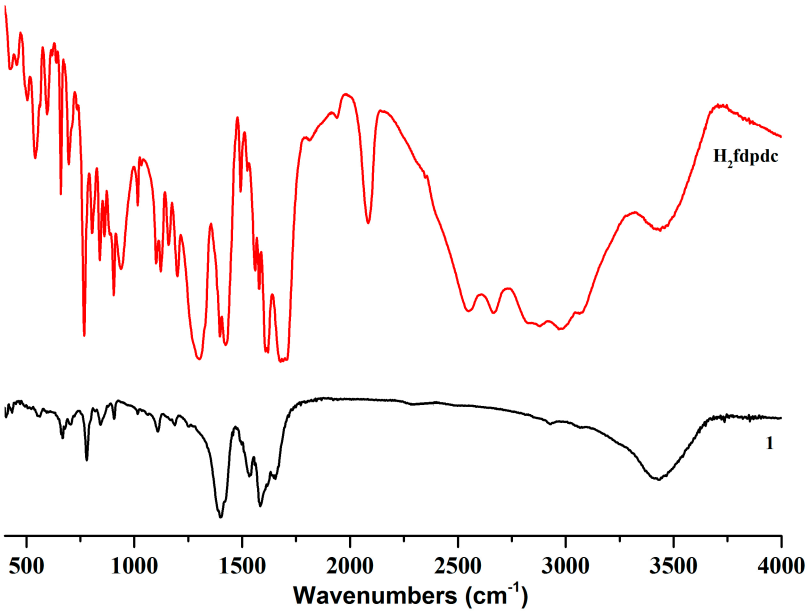

Figure S1. IR spectrum of **1** and H_2fdpdc.

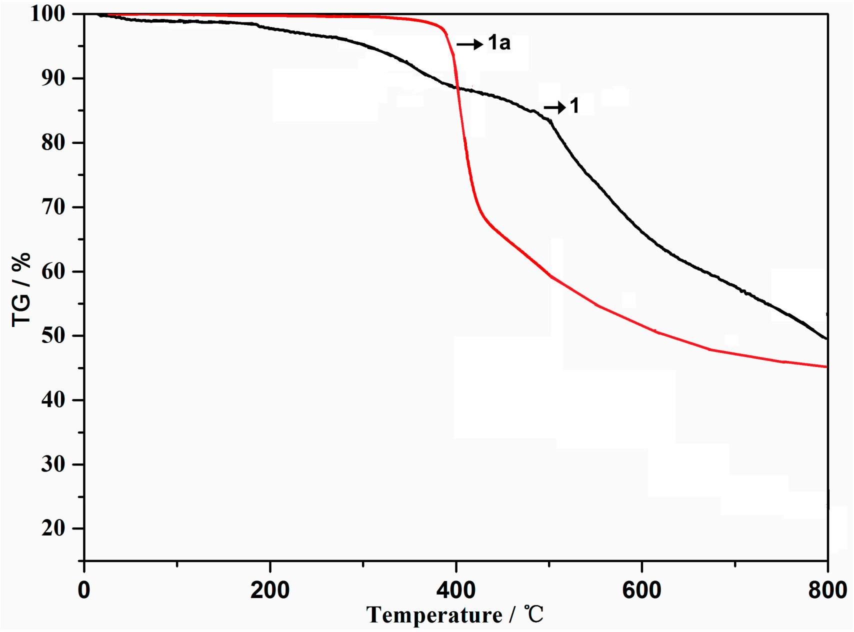

Figure S2. TGA curves of **1** and activated **1** (**1a**).

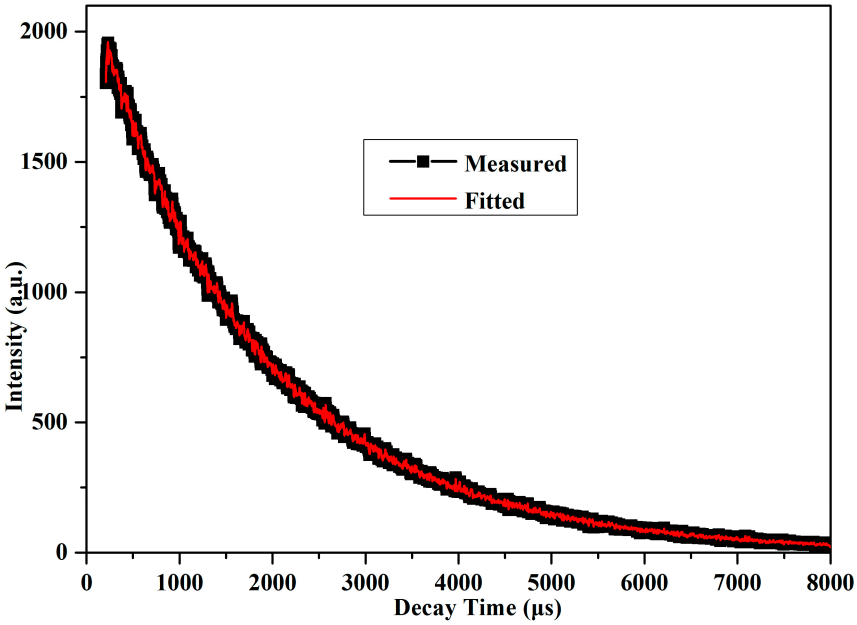

Figure S3. Luminescent lifetime for **1** in solid.
